# Molecular characterization of H6 subtype influenza viruses in southern China from 2009 to 2011

**DOI:** 10.1038/emi.2016.71

**Published:** 2016-07-20

**Authors:** Shumei Zou, Rongbao Gao, Ye Zhang, Xiaodan Li, Wenbing Chen, Tian Bai, Libo Dong, Dayan Wang, Yuelong Shu

**Affiliations:** 1 National Institute for Viral Disease Control and Prevention, Collaboration Innovation Center for Diagnosis and Treatment of Infectious Diseases, Chinese Center for Disease Control and Prevention, Key Laboratory for Medical Virology, National Health and Family Planning Commission, Beijing 102206, PR China

**Keywords:** H6 avian influenza virus, molecular characterization, phylogenetic analysis, southern China

## Abstract

H6 avian influenza viruses (AIVs), which are prevalent in domestic and wild birds in Eurasian countries, have been isolated from pigs, a dog and a human. Routine virological surveillance at live poultry markets or poultry farms was conducted in southern China from 2009 to 2011. This study investigated the genetic and antigenic characteristics, analyzed the receptor-binding properties and evaluated the kinetics of infectivity of the AIVs in A549, MDCK and PK15 cells. A total of 14 H6N6 and 2 H6N2 isolates were obtained from four provinces in southern China. Genetic analysis indicated two distinct hemagglutinin lineages of the H6 strains cocirculating in southern China, and these strains facilitated active evolution and reassortment among multiple influenza virus subtypes from different avian species in nature. None of these isolates grouped with the novel Taiwan H6N1 virus responsible for human infection. Receptor-binding specificity assays showed that five H6 AIVs may have acquired the ability to recognize human receptors. Growth kinetics experiments showed that EV/HB-JZ/02/10(H6N2) and EV/JX/15/10(H6N6) initially reproduced faster and achieved higher titers than other viruses, suggesting that enhanced binding to α-2,6-linked sialic acids correlated with increased viral replication in mammalian cells. Overall, the results emphasize the need for continued surveillance of H6 outbreaks and extensive characterization of H6 isolates to better understand genetic changes and their implications.

## INTRODUCTION

Extensive investigations have revealed that influenza viruses could infect a wide range of hosts, including many types of birds, human, pigs, horses, dogs, cats and other mammals.^1^ Of these species, aquatic birds are considered a natural reservoir of avian influenza viruses (AIVs).^2^ For a long time, worldwide AIVs control and surveillance efforts have focused on the H5, H7 and H9 subtypes because of the high mortality or the significant economic losses these subtypes caused in affected flocks. However, it is difficult to predict which subtype of AIV might be able to cross the species barrier and infect humans in the future. Increasing evidence demonstrates that low-pathogenic avian influenza viruses (LPAI) are also important, primarily because they could potentially infect domestic poultry and humans if they undergo reassortment to produce pathogenic forms.^3, 4^ This possibility was demonstrated in 2013 by the unexpected emergence of the LPAI H7N9 virus in humans in China; H7N9 has caused over 700 confirmed human infections, with fatality ~39%.^5^

The first H6 influenza virus was isolated from a turkey in 1965, since then, H6 viruses have been isolated with increasing frequency from wild, domestic aquatic and terrestrial avian species throughout the world. At present, H6 AIVs have a broader host range than any other subtype AIVs,^6^ and have been one of the most commonly recognized subtypes in domestic ducks in southern China.^7, 8^ Previous studies suggested that H6 AIVs were involved in the generation of human H5N1, H9N2 and H5N6 viruses.^9, 10^

The H6 subtype reassortant viruses might have crossed the species barrier and infected mammals, including humans, without adaptation. For example, in southern and eastern China, H6N6 AIVs were isolated from swine with clinical signs,^11, 12^ and in Taiwan, an H6N1 virus was isolated from a dog.^13^ Recent seroprevalence research showed seropositivity for H6 viruses among occupational exposure workers in 19 provinces of China,^14^ and veterinarians exposed to birds showed H6-specific antibody was significantly elevated in United States.^15^ In May 2013, an H6N1 virus was isolated in Taiwan from a 20-year-old woman with symptoms including fever, cough, headache and muscle ache.^16^ The study of H6 viruses isolated from Taiwan in the past 14 years suggests an elevated threat of H6 viruses to human health.^17^ These data indicate that H6 subtype AIVs might pose a potential public health threat.

In this study, we investigated influenza H6 subtype viruses isolated in mainland China for their genetic and antigenic characteristics, the receptor-binding properties and kinetics of infectivity in mammalian cells.

## MATERIALS AND METHODS

### Virus isolation and identification

The H6 viruses used in this study were isolated from environmental samples collected from live poultry markets (LPMs) or poultry farms in China according to the guideline of AIVs surveillance of Chinese Center for Disease Control and Prevention in live poultry related environments. Viruses were isolated in specific pathogen-free chicken embryonated eggs at 37 °C for 48 h. Allantoic fluid was harvested and hemagglutination (HA) assay with 1% turkey red blood cells (TRBCs) was used to confirm the presence of influenza viruses. The HA positive samples were further subtyped by reverse transcription-polymerase chain reaction (RT-PCR) using 16 sets of HA (H1–H16) primers and nine sets of neuraminidase (NA) (N1–N9) primers designed by the Chinese National Influenza Center.

### Viral RNA extraction and RT-PCR

Virus RNA was extracted from allantoic fluid using the RNeasy Mini kit (Qiagen, Hilden, Germany) and was transcribed into complimentary DNA (cDNA) using the Uni12 primer (5′-AGC AAA AGC AGG-3′) and SuperRT cDNA kit (CWBIO, Beijing, China) according to the manufacturers' recommendations. cDNAs of eight segments were then amplified with gene specific primers designed by the Chinese National Influenza Center (primer sequences are available upon request). The PCR reaction contained 2 μL cDNA, 1 μL forward primer and reverse primer, 12.5 μL 2 × EsTaq MasterMix (CWBIO, Beijing, China) and 8.5 μL RNase-free water with a final volume of 25 μL. A single PCR program was used for all primers, i.e., initial denaturation at 94 °C for 2 min, 35 cycles of denaturation at 94 °C for 30 s, annealing at 55 °C for 30 s, and extension at 72 °C for 1 min and 30 s.

### Genome sequencing and phylogenetic analysis

The PCR products were visualized by agarose gel electrophoresis and then purified with the QIAquick PCR purification kit (Qiagen, Hilden, Germany). The purified PCR products were then sequenced using the BigDye Terminator v3.1 Cycle Sequencing Kit (Applied Biosystems, Foster, CA, USA) and analyzed with an ABI 3730 DNA analyzer (Applied Biosystems, Foster City, CA, USA) according to the manufacturer's instructions. Full genome sequences of the viruses were deposited in the Global Initiative on Sharing Avian Influenza Data (GISAID) database (Accession NO EPI717857-717984). Phylogenetic trees were generated using the neighbor-joining method with MEGA software (version 5.01, Molecular Evolutionary Genetics Analysis (MEGA); http://megasoftware.net/), and the bootstrap value was tested with 1000 replications for each gene.

### Receptor-binding analysis by HA assay

A HA assay using 1% TRBC, which was treated with α-2,3-specific sialidase, was performed as previously described^18^ with minor modifications. Briefly, 10% TRBC suspension prepared in phosphate buffer solution was treated by 625 mU 2,3-specific sialidase (Takara, Dalian, China) at 37 °C for 30 min. Complete elimination of the α-2,3-receptor on sialidase-treated TRBCs was confirmed by receptor staining and flow cytometry. These 1% TRBCs or 1% sialidase-treated TRBCs were used in the HA assay. The pdm09H1N1 virus A/California/04/2009 (H1N1) and RG-A/Anhui/1/2005(H5N1) were used as controls for the HA test.

### Antigenic analyses

The antigenic characteristics of the H6 influenza viruses were analyzed by hemagglutination inhibition (HI) assays with post-infection ferret anti-sera raised against A/environment/Jiangxi/25/2009, A/environment/Hunan-Changsha/2/2010 and A/Taiwan/2/2013, and hyperimmune rabbit anti-sera against A/wild waterfowl/Dongting/C2638/2011 and A/wild waterfowl/Dongting/C2029/2011, which were kindly provided by Dr Yun Zhu.^19^ The HI assays were performed as previously described.^19^ Briefly, before testing in the HI assay, serum samples were treated with a 1:4 (vol/vol) of receptor destroying enzyme (RDE) at 37 °C for 18 h followed by incubation at 56 °C for 30 min. Serum samples were titrated in twofold dilutions in phosphate buffer solution and tested at an initial dilution of 1:10. Virus with a concentration of 4 HAU/25 μL was used for the HI tests.

### Viral replication kinetics in A549, MDCK and PK15 cells

To analyze the capability of viral replication in mammary cells, we selected four viruses (based on their receptor-binding properties: EV/JX/24/09(H6N6) and EV/GX/02/10(H6N2) only bound to α-2,3-receptors; EV/HB-JZ/02/10(H6N2) and EV/JX/15/10(H6N6) could bind α-2,6 receptors) to infect human-type II alveolar epithelial (A549), Madin–Darby canine kidney (MDCK) and porcine kidney (PK15) cells. These cells were obtained from the American Type Culture Collection. Briefly, the selected viruses were inoculated into A549, MDCK or PK15 monolayers at a multiplicity of infection of 0.01. The inoculated cells were incubated with DMEM containing 0.5% BSA and 2 μg/mL TPCK-treated trypsin (Sigma-Aldrich, St Louis, MO, USA) at 37 °C. The supernatants were collected at 12, 24, 36, 48 and 60 h post of infection, respectively, and stored at −80 °C for viral titration. The viral titers of these supernatants were determined by end-point titration in MDCK cells as described previously.^20^ The viral replication titers were compared for the different viruses, and statistical significance was determined by nonparametric *t*-tests using GraphPad (Vision 5.0, GraphPad Prism). Differences were considered significant at *P*<0.05 with two-tailed testing.

## RESULTS

### Genetic analysis of the HA and NA genes

A total of 14 H6N6 and 2 H6N2 isolates were obtained from four provinces in southern China during 2009–2011 (Table 1). The full-length genome sequences of these viruses were sequenced and analyzed. The homological analysis showed that the HA gene of EV/HB-JZ/02/10(H6N2) shared a low similarity (86.8%–89.5%) with others, and the other 15 viruses have a high nucleotide identity among each other (93.8%–99.9%). In addition, the HA genes of the 16 isolates presented a low nucleotide identity ranging from 83.7% to 89.1% with the human isolate A/Taiwan/2/2013(H6N1) (TW/2/13).

**Figure 1:**
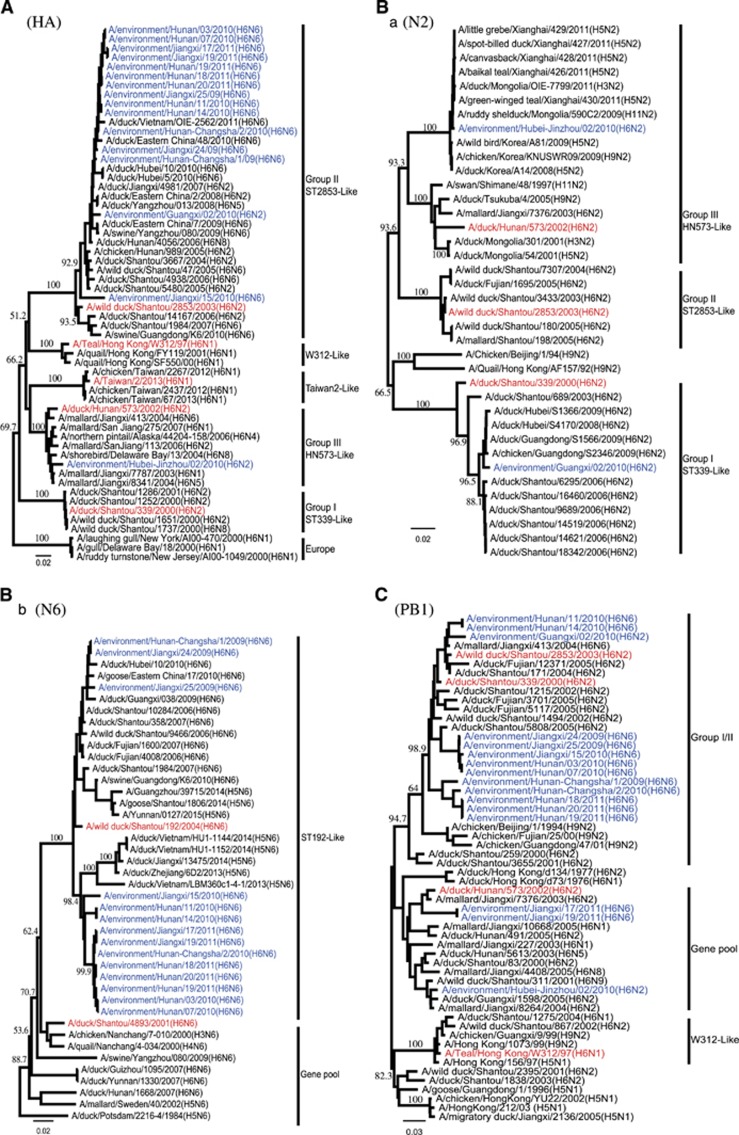
Phylogenetic trees of HA (**A**), N2 NA (**B-a**), N6 NA (**B-b**), PB1 (**C**) genes of H6 subtype AIVs. Full-length sequences with complete open reading frames were used for the phylogenetic analyses, and neighbor-joining (NJ) trees were generated using MEGA 5.01. Estimates of the phylogenies were calculated by performing 1000 neighbor-joining bootstrap replicates. The phylogenetic tree of the HA and N2 NA trees were rooted to A/turkey/Canada/63 (H6N2), and the N6 NA tree was rooted to A/duck/England/1956 (H11N6). PB1 was rooted to A/pintail duck/Alberta/628/1979 (H6N8). Our 16 isolates are highlighted in blue, and representative strains are shown in red.

In a previous study, H6 subtype viruses were divided into three major groups based on the HA gene (I, II and III/gene pool).^7, 8^ All 16 H6 HA genes in this study belonged to either group II or III; 15 viruses clustered together and belonged to group II, which contained the H6N2, H6N5, H6N6 and H6N8 viruses that were isolated from southern China from 2004 to 2011. Two H6N6 strains isolated previously from pigs also belonged to this group,^11, 12^ suggesting the sharing of a common HA gene origin. Only a single isolate (EV/HB-JZ/02/10) grouped with previously described group III (gene pool), which was composed of viruses isolated from poultry, wild or migratory birds (Figure 1A). Viruses of group III included the biggest diversity of NA subtype combinations, including N1, N2, N4, N5 and N8. None of the viruses in this study grouped with the group I and human-source TW/2/13-like viruses.

Phylogenetic analysis of the N6 genes showed that all N6 NA genes clustered together to form a major lineage and clustered with the A/wild duck/Shantou/192/2004 (ST192-like) H6N6 viruses circulating among domestic poultry in Fujian and Guangdong provinces (Table 2 and Figure 1B and b).

Phylogenetic analysis of the N2 gene showed that EV/HB-JZ/02/10 and EV/GX/02/10 belonged to two separate groups. The NA of EV/GX/02/10 belonged to the A/duck/shantou/339/2000 (ST/339/00-like) lineage in a previous report. The NA of EV/HB-JZ/02/10 belonged to the A/duck/hunan/573/2002 (HN/573/02-like) lineage (Figure 1B and a), which included H1, H2, H3, H4, H5, H6, H7, H9, H11 subtype combinations.

### Genetic analysis of the internal genes

Phylogenetic analysis of the internal genes indicated that these isolates were generated from genetic reassortment of viruses of different lineages. The majority of the H6 isolates reassorted with an NP gene from the group III, except EV/HB-JZ/02/10, which NP belonged to the group I/II lineage. On the contrary, from the phylogenetic trees of the PB2, MP and NS genes, 15 H6 viruses clustered together into group I/II, except EV/HB-JZ/02/10, which was most closely related to the group III. Phylogenetic analyses of the PB1 and PA genes showed that EV/JX/17/11 and EV/JX/19/11 obtained PB1 from group III (Figure 1C), and EV/GX/02/10 obtained PA genes from group III. Therefore, exchanges between the gene pool and the group I/II internal genes occurred more frequently for the NP, PB1 and PA genes, with a few exchanges involving the remaining genes (Table 2).

### Molecular characterization

On the basis of this analysis, the HA genes of most H6 viruses had an open reading frame of 1701 bp that encoded a precursor polypeptide of 566 amino acids. EV/JX/15/10(H6N6) had an open reading frame of 1695 bp, which has a 6-nucleotide deletion at positions 515 to 520 that results in the loss of two amino acids at position 172 to 173. All the H6 isolates possessed the sequence PQIETR↓GLF at the cleavage site between HA1 and HA2; this sequence is also commonly observed in H6 viruses from terrestrial poultry. None of the isolates contained a sequence with multiple basic amino acids (Table 3), which are found in highly pathogenic avian influenza viruses (HPAI).^21^ The amino acid changes of A138S (H3 numbering) were present in EV/JX/15/10 and also reported in Swine/GD/K6/10 (H6N6). The consensus amino acid sequences revealed that the majority of H6 viruses had six potential N-linked glycosylation sites including four in HA1 (26 or 27, 39, 306 and 311) and two in HA2 (498 and 557). Except the HA protein of EV/HB-JZ/02/10, the other viruses presented a substitution of N182R/T in HA, which led to the loss of an N-glycosylation site.

The NAs of the H6 isolates had no amino acid deletions in the stalk region, except for two H6N6 isolates (EV/JX/24/09 and EV/HN/1/09), which had a 27-nucleotide deletion at the NA stalk region of residues 177 to 203, which results in the loss of nine amino acids at position 60 to 68. This amino acid deletion might be associated with increased virulence in mammals.^22^ In addition, mutations E119V, H275Y, R293K and N295S were not detected in the NA protein, suggesting the isolates are sensitive to neuraminidase inhibitors such as oseltamivir.^23^ The S31N mutation responsible for amantadine resistance was not found in the M2 protein. No amino acid mutations associated with increased virulence in mammals were detected in the PB1, PB2 or NS proteins (Table 3).

### Receptor-binding specificity of H6 avian influenza viruses

Recently, an α-2,3-specific sialidase-treated red blood cell (RBC) HA assay was developed and used for receptor specificity screening.^18, 24^ The α-2,6 or α-2,3-binding preference could be distinguished by the change of HA titer reacted with RBCs and enzymatic RBCs. Herein, we analyzed the receptor-binding specificity of the H6 isolates using HA assays with sialidase-treated TRBCs, which have only α-2,6 receptors. Theoretically, sialidase digestion should abolish the HA titers by α-2,3-specific viruses, whereas viruses that could bind to α-2,6 receptors should maintain HA activity with the treated TRBCs. We found that five out of 16 H6 viruses agglutinated the sialidase-treated-TRBCs. The HA titers are shown in Table 4. Compared with untreated TRBCs, five H6 viruses (including four H6N6 and one H6N2) still show HA activity with α-2,3-sialidase-treated TRBCs, which had only α-2,6 receptors, whereas RG-A/Anhui/1/2005(H5N1) and A/California/04/2009(H1N1)viruses were preferentially bound to α-2,3 and α-2,6 receptors, respectively, as expected. Our results indicated these H6 viruses could bind human-type (α-2,6) receptors. The H6 AIVs circulating in the LPMs of southern China might have acquired the ability to recognize the human-type receptors.

### Antigenic analysis

A previous study^7, 8^ reported that H6 AIVs underwent significant antigenic changes since 1997. To determine if antigenic changes have occurred, we characterized the antigens of the H6 isolates using HI tests. None of the tested H6 viruses reacted well with ferret anti-sera raised from TW/2/13(H6N1), suggesting all H6 isolates were antigenically distinguishable from TW/2/13-like viruses.

The group II viruses had no or low reactivity to anti-DT/C2638/11 and anti-DT/C2019 /11 (group III). EV/HB-JZ/02/10, which belongs to group III, failed to react with anti-EV/JX/25/09 and anti-EV/HN/2/10 (group II) (Table 5). These findings demonstrate the antigenic differences between the H6 viruses prevailing in domestic poultry in southern China.

### Growth kinetics of H6 viruses in MDCK, A549 and PK15 cells

**Figure 2:**
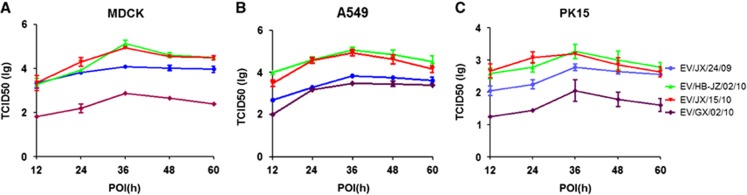
Viral replication kinetics in mammary cells. Monolayers were inoculated at an multiplicity of infection of 0.01 with each virus, and the culture supernatants were collected at the indicated time points and titrated in MDCK. The curves display the multicycle replication titers of H6 viruses in MDCK (**A**), A549 (**B**) or PK15 (**C**) cells. The results are shown as the means±standard deviations of triplicate samples. Abbreviation: Madin–Darby canine kidney, MDCK.

To further investigate the replication capacity of the H6 AIVs in mammalian cells, the kinetics of infectivity were determined by multiple-cycle growth curves of four selected viruses in A549, MDCK and PK15 cells. The results showed that the four H6 viruses grew efficiently and reached a peak level at 36 h post infection. However, EV/GX/02/10 showed significantly lower titers than the other viruses in MDCK, as shown in Figure 2A; the mean peaking titers of EV/JX/24/09, EV/HB-JZ/02/10, EV/JX/15/10 and EV/GX/02/10 were 10^4.08^, 10^5.14^, 10^4.94^ and 10^2.87^ TCID50/100 μL, respectively (*P*<0.01). EV/HB-JZ/02/10 and EV/JX/15/10 had similar replication kinetics and displayed a higher replication level than the other two H6 viruses in A549 cells, as shown in Figure 2B; the mean peaking titers of EV/JX/24/09, EV/HB-JZ/02/10, EV/JX/15/10 and EV/GX/02/10 were 10^3.83^, 10^5.08^, 10^4.95^ and 10^3.5^ TCID50/100 μL, respectively (*P*<0.05). In PK15 cells, EV/HB-JZ/02/10 and EV/JX/15/10 also showed higher titers than the other two viruses, and the mean peaking titers of EV/JX/24/09, EV/HB-JZ/02/10, EV/JX/15/10 and EV/GX/02/10 were 10^2.78^, 10^3.28^, 10^3.2^ and 10^2.06^ TCID50/100 μL, respectively (Figure 2C). In addition, all the viral titers were significantly lower than those in the infected MDCK or A549 cells. Taken together, these data indicated that EV/HB-JZ/02/10 and EV/JX/15/10, which could bind α-2,6 receptors, were able to replicate at higher levels in mammary cells.

## DISCUSSION

The Asian HPAI H5N1 virus, two LPAI H9N2 (G1 and Y280) lineages, and H7N9 virus are considered to be pandemic threats.^4^ The H6 viruses have been cocirculating with these viruses for years, there is a risk for H6 influenza viruses to promote interactions or gene exchanges among these viruses.^25, 26^ Therefore, H6 AIVs raise a potential threat to public health, and continued surveillance is becoming essential.

The results of the present study indicated that at least two H6 subtypes (H6N2 and H6N6) were cocirculating in southern China. In our study, H6N6 was the most prevalent subtype. The H6 HA gene tree clearly demonstrated that the majority of the H6 viruses cluster together and belong to the group II lineage, except EV/HB-JZ/02/10 (grouped with group III). All these H6 viruses were different from the W312-like^9^ and ST339-like viruses and were different from the TW/2/13-like viruses; the H6 viruses have undergone extensive reassortment. Phylogenetic analysis of the N2 genes showed that these viruses formed two distinct lineages; most viruses clustered to group I/II, which incorporates N2 NAs and continues to prevail in eastern and southern China, whereas EV/HB-JZ/02/10 might have acquired its N2 gene segments from wild birds.^7^ All the NA genes of the N6 viruses from this study clustered together to form a major lineage composed of the H6N6 viruses isolated from Shantou and Fujian province. The results revealed that group II H6 viruses continued to dominate in southern China, and it seems that N6 is more common than N2.

Phylogenetic analyses of the internal genes revealed that multiple reassortments or gene exchanges had occurred, and the gene exchanges between the gene pool and the group I/II occurred more frequently for the NP, PB1 and PA genes, with a few exchanges involving the remaining genes. It is noteworthy that EV/HB-JZ/02/10 belonging to the group III appeared to have donated genes for the generation of variant subtype AIVs, such as H5, H7, H9 and H10. The H6 viruses from group III contributed to the genetic diversity and complexity of influenza virus ecology in southern China. Phylogenetic analysis also revealed that multiple transmissions or gene mixing already occurred between migratory waterfowl and domestic birds. It was not clear if these H6 viruses were introduced into backyard ducks from wild birds or transmitted back to aquatic birds in reverse. However, there is a possibility that wild birds were the source of these viruses because the H6 viruses were the most abundantly detected viruses in wild birds.^27, 28, 29^ Future studies are needed to elucidate gene precursors and to better understand the ecology and evolution of the influenza virus in southern China.

It is generally accepted that HA receptor-binding preference to a-2,6-linked (human-type receptors) sialylated glycans is the initial key step for a novel influenza-virus-causing pandemic.^30, 31^ Binding to α-2,6-linked sialic acids (SA) is a prerequisite for AIVs to transmit from human to human.^32, 33, 34^ In the present study, we found that five H6 strains derived from the environment still showed HA activity with TRBCs, which had only α-2,6 receptors, indicating the contemporary H6 viruses could bind to the α-2,6-linked SA and might acquire the ability to recognize human-type receptors. A recent study also found that 34% of the 257 H6 viruses isolated in southern China between 2008 and 2011 recognized the human-type receptor.^35^ Different subtypes of HA proteins seem to have different sequence requirements for avian to human receptor switching.^36^ For H1 HA, residues at HA1 190 and 225 determine receptor specificity.^37, 38, 39^ However, for H2 and H3 HA, the proteins L226 and S228 are for the human receptor and Q226 and G228 are for the avian receptor.^40, 41^ For H5 HA, two more amino acid mutations, 226L/228S or 224N/226L, combined with the absence of glycosylation at positions 158 to 160 in HA, enable the H5N1 influenza viruses to preferentially recognizeα-2,6-linked SA.^42, 43, 44, 45^ For H7 HA, the mutations Q226L and G228S, separately or combined, enhance the binding to human receptors.^46, 47^ The H6 HA G228S change was reported in the human H6 virus isolates; the combination of residues at HA1 N137, V190 and S228 of TW/2/13 H6 HA might enhance binding affinity for human-like receptors and slightly weakened binding for avian-like receptors.^17^ None of our H6 viruses that bound to α-2,6-linked SA have the 226L/228S and other any amino acid changes in their HA that associated with the human virus-like binding properties, and the amino acid composition of HA1 S137, E190, and G228 in the receptor-binding site allows binding to both human- and avian-like receptors; the result was with same as reported in a previous study.^17^ Therefore, the key amino acid mutations in HA responsible for the affinity of H6 viruses to the human-like receptor deserve further investigation. The overall binding of H6 HAs to both avian and human-like receptors tested in this study justifies the need for diligent surveillance of H6 viruses around the world with particular attention to southern China isolates.

Previous studies showed that the H6 viruses from wild birds or LPMs replicated efficiently in MDCK or A549 cells;^35, 48^ in addition, the virus with preferential binding to α-2,6- receptors might have contributed to its high level of replication in A549 cells. In our study, *in vitro* growth kinetics revealed that EV/HB-JZ/02/10(H6N2) and EV/JX/15/10(H6N6) initially grew faster and achieved higher titers than the other viruses. These data also showed that the enhanced binding to α-2,6-linked SA correlated with increased viral replication in mammalian cells. However, the NA protein of EV/JX/24/09(H6N6) had nine amino acid (position 60 to 68) deletions in the stalk region, which may be associated with increased viral replication *in vitro*.

## Figures and Tables

**Table 1:** H6 viruses characterized in this study

**Virus**	**Subtype**	**Abbreviation**	**Isolation date**	**Location of isolation**	**Accession number**
A/environment/Jiangxi/24/2009	H6N6	EV/JX/24/09	07/2009	Jiangxi	EPI717857-64
A/environment/Jiangxi/25/2009	H6N6	EV/JX/25/09	07/2009	Jiangxi	EPI717865-72
A/environment/Hunan-Changsha/1/2009	H6N6	EV/HN/1/09	04/2009	Changsha	EPI717873-80
A/environment/Hubei-Jinzhou/02/2010	H6N2	EV/HB-JZ/02/10	10/2010	Jinzhou	EPI717881-88
A/environment/Hunan-Changsha/2/2010	H6N6	EV/HN/2/10	12/2010	Changsha	EPI717889-96
A/environment/Hunan/03/2010	H6N6	EV/HN/03/10	11/2010	Hunan	EPI717897-717904
A/environment/Hunan/07/2010	H6N6	EV/HN/07/10	11/2010	Hunan	EPI717905-12
A/environment/Jiangxi/15/2010	H6N6	EV/JX/15/10	12/2010	Jiangxi	EPI717913-20
A/environment/Hunan/11/2010	H6N6	EV/HN/11/10	10/2010	Hunan	EPI717921-28
A/environment/Hunan/14/2010	H6N6	EV/HN/14/10	10/2010	Hunan	EPI717929-36
A/environment/Guangxi/02/2010	H6N2	EV/GX/02/10	7/2010	Guangxi	EPI717937-44
A/environment/Jiangxi/17/2011	H6N6	EV/JX/17/11	3/2011	Jiangxi	EPI717945-52
A/environment/Jiangxi/19/2011	H6N6	EV/JX/19/11	3/2011	Jiangxi	EPI717953-60
A/environment/Hunan/18/2011	H6N6	EV/HN/18/11	7/2011	Hunan	EPI717961-68
A/environment/Hunan/19/2011	H6N6	EV/HN/19/11	11/2011	Hunan	EPI717969-76
A/environment/Hunan/20/2011	H6N6	EV/HN/20/11	11/2011	Hunan	EPI717977-84

**Table 2:** Gene constellation of H6 viruses isolated from the environment in southern China

**Virus**^a^	**Gene segment**^b^							
	**HA**	**NA**^c^	**PB2**	**PB1**	**PA**	**NP**	**MP**	**NS**
EV/JX/24/09	Group II	ST192	Group I/II	Group I/II	Group I/II	Group III	Group I	Group I
EV/JX/25/09	Group II	ST192	Group I/II	Group I/II	Group I/II	Group III	Group I	Group I
EV/HN/1/09	Group II	ST192	Group I/II	Group I/II	Group I/II	Group III	Group I	Group I
EV/HB-JZ/02/10	Group III	Group III	Group III	Group III	Group III	Group I/II	Group III	Group III
EV/HN/2/10	Group II	ST192	Group I/II	Group I/II	Group I/II	Group III	Group I	Group I
EV/HN/03/10	Group II	ST192	Group I/II	Group I/II	Group I/II	Group III	Group I	Group I
EV/HN/07/10	Group II	ST192	Group I/II	Group I/II	Group I/II	Group III	Group I	Group I
EV/JX/15/10	Group II	ST192	Group I/II	Group I/II	Group I/II	Group III	Group I	Group I
EV/HN/11/10	Group II	ST192	Group I/II	Group I/II	Group I/II	Group III	Group I	Group I
EV/HN/14/10	Group II	ST192	Group I/II	Group I/II	Group I/II	Group III	Group I	Group I
EV/GX/02/10	Group II	Group I	Group I/II	Group I/II	Group III	Group III	Group I	Group I
EV/JX/17/11	Group II	ST192	Group I/II	Group III	Group I/II	Group III	Group I	Group I
EV/JX/19/11	Group II	ST192	Group I/II	Group III	Group I/II	Group III	Group I	Group I
EV/HN/18/11	Group II	ST192	Group I/II	Group I/II	Group I/II	Group III	Group I	Group I
EV/HN/19/11	Group II	ST192	Group I/II	Group I/II	Group I/II	Group III	Group I	Group I
EV/HN/20/11	Group II	ST192	Group I/II	Group I/II	Group I/II	Group III	Group I	Group I

a The full names and subtypes of these viruses are listed in Table 1.

b Genotypes were established in light of the phylogenetic relationships; Group I/II: groups I and II.

c N6 lineage represented by wild duck/Shantou/192/2004(ST192-like), which was described in a previous study.^8^

**Table 3:** Molecular characteristics of H6 viruses in this study

**Virus**	**Amino acid sequence at cleavage site of HA**	**Receptor-binding sites in HA**^a^						**Key position in HA**^a^			**Amino acid deletion in NA (position)**	**M2**	**PB2**		**NS1**	**PA**
		**137**	**138**	**169–171**	**186**	**190**	**226–228**	**156**	**263**	**380**		**31**	**627**	**701**	**92**	**38**
EV/JX/24/09	PQIETR↓GLF	S	A	TNT	P	E	QRG	H	S	N	Yes (60–68)	S	E	D	D	I
EV/JX/25/09	PQIETR↓GLF	S	A	RNT	P	E	QRG	H	S	N	No	S	E	D	D	I
EV/HN/1/09	PQIETR↓GLF	S	A	TNT	P	E	QRG	H	S	N	Yes (60–68)	S	E	D	D	I
EV/HB-JZ/02/10	PQIETR↓GLF	K	A	NNT	P	E	QRG	H	N	N	No	S	E	D	D	I
EV/HN/2/10	PQIETR↓GLF	S	A	RNT	P	E	QRG	H	S	N	No	S	E	D	D	I
EV/HN/03/10	PQIETR↓GLF	S	A	RNT	P	E	QRG	H	S	N	No	S	E	D	D	I
EV/HN/07/10	PQIETR↓GLF	S	A	RNT	P	E	QRG	H	S	N	No	S	E	D	D	I
EV/JX/15/10	PQIETR↓GLF	S	S	RNT	T	E	QRG	R	N	N	No	S	E	D	D	I
EV/HN/11/10	PQIETR↓GLF	S	A	RNT	P	E	QRG	H	S	N	No	S	E	D	D	I
EV/HN/14/10	PQIETR↓GLF	S	A	RNT	P	E	QRG	H	S	N	No	S	E	D	D	I
EV/GX/02/10	PQIETR↓GLF	S	A	TNT	P	E	QRG	H	S	N	No	S	E	D	D	I
EV/JX/17/11	PQIETR↓GLF	S	A	RNT	P	E	QRG	H	S	N	No	S	E	D	D	I
EV/JX/19/11	PQIETR↓GLF	S	A	RNT	P	E	QRG	H	S	N	No	S	E	D	D	I
EV/HN/18/11	PQIETR↓GLF	S	A	RNT	P	E	QRG	H	S	N	No	S	E	D	D	I
EV/HN/19/11	PQIETR↓GLF	S	A	RNT	P	E	QRG	H	S	N	No	S	E	D	D	I
EV/HN/20/11	PQIETR↓GLF	S	A	RNT	P	E	QRG	H	S	N	No	S	E	D	D	I

a H3 numbers were used throughout.

**Table 4:** Receptor specificity of H6 viruses with TRBC and α2,3-sialidase-treated TRBCs

**Virus**	**HA titers with untreated 1% TRBC (HAU/50 μL)**	**HA titers**^a^ **with sialidase-treated 1% TRBC (HAU/50 μL)**	**Receptor specificity**
EV/JX/24/09	64	Neg	α2,3-SA binding
EV/JX/25/09	32	8	α2,3-SA and α2,6-SA binding
EV/HN/1/09	256	Neg	α2,3-SA binding
EV/HB-JZ/02/10	128	32	α2,3-SA and α2,6-SA binding
EV/HN/2/10	64	Neg	α2,3-SA binding
EV/HN/03/10	64	Neg	α2,3-SA binding
EV/HN/07/10	16	Neg	α2,3-SA binding
EV/JX/15/10	64	64	α2,3-SA and α2,6-SA binding
EV/HN/11/10	32	Neg	α2,3-SA binding
EV/HN/14/10	32	Neg	α2,3-SA binding
EV/GX/02/10	16	Neg	α2,3-SA binding
EV/JX/17/11	32	8	α2,3-SA and α2,6-SA binding
EV/JX/19/11	64	16	α2,3-SA and α2,6-SA binding
EV/HN/18/11	32	Neg	α2,3-SA binding
EV/HN/19/11	32	Neg	α2,3-SA binding
EV/HN/20/11	32	Neg	α2,3-SA binding
RG-A/anhui/1/05^b^	512	Neg	α2,3-SA binding
A/CA/04/09^b^	64	64	α2,6-SA binding

a Neg: no HA titer, negative.

b The full names and subtypes of the viruses are described in ‘Materials and Methods' section.

**Table 5:** Antigenic analysis of H6 viruses by HI

**Virus**	**Titers**^a^ **with the indicated antiserum (HA group)**				
	**EV/JX/25/09 (II)**	**EV/HN/2/10 (II)**	**DT/C2638/11 (III)**	**DT/C2029/11 (III)**	**TW/02/13**
*Group II*					
EV/JX/25/09	**320**	640	<10	40	<10
EV/HN/2/10	320	**320**	<10	20	<10
EV/JX/24/09	1280	640	<10	80	<10
EV/HN/1/09	640	1280	10	160	<10
EV/HN/03/10	320	320	<10	20	<10
EV/HN/07/10	640	640	<10	160	<10
EV/JX/15/10	320	320	<10	10	<10
EV/HN/11/10	320	320	<10	40	<10
EV/HN/14/10	640	640	<10	20	<10
EV/GX/02/10	320	640	<10	80	<10
EV/JX/17/11	640	640	<10	40	<10
EV/JX/19/11	320	640	<10	40	<10
EV/HN/18/11	320	320	<10	40	<10
EV/HN/19/11	320	320	<10	40	<10
EV/HN/20/11	160	160	<10	10	<10
					
*Group III*					
DT/C2638/11^b^	<10	<10	160	320	<10
DT/C2029/11^b^	<10	<10	40	320	<10
EV/HB-JZ/02/10	<10	<10	320	1280	<10
					
*TW-like*					
TW/02/13^b^	10	10	40	40	**1280**

a Homologous titers are bold; <10: no inhibition was detected at a serum dilution of 1:10.

b The full names and subtypes of the viruses are described in ‘Materials and Methods' section.
